# The Role of Macrophages in Atherosclerosis: Pathophysiologic Mechanisms and Treatment Considerations

**DOI:** 10.3390/ijms24119568

**Published:** 2023-05-31

**Authors:** Panagiotis Theofilis, Evangelos Oikonomou, Konstantinos Tsioufis, Dimitris Tousoulis

**Affiliations:** 1 First Department of Cardiology, “Hippokration” General Hospital, University of Athens Medical School, 11527 Athens, Greecektsioufis@med.uoa.gr (K.T.); 2 Third Department of Cardiology, Thoracic Diseases General Hospital “Sotiria”, University of Athens Medical School, 11527 Athens, Greece

**Keywords:** atherosclerosis, inflammation, macrophage, pathophysiology

## Abstract

Atherosclerotic diseases are a leading cause of morbidity and mortality worldwide, despite the recent diagnostic and therapeutic advances. A thorough understanding of the pathophysiologic mechanisms is thus essential to improve the care of affected individuals. Macrophages are crucial mediators of the atherosclerotic cascade, but their role has not been fully elucidated. The two main subtypes, tissue-resident and monocyte-derived macrophages, have distinct functions that contribute to atherosclerosis development or regression. Since polarization of macrophages to an M2 phenotype and induction of macrophage autophagy have been demonstrated to be atheroprotective, targeting these pathways could represent an appealing approach. Interestingly, macrophage receptors could act as drug targets, as seen in recent experimental studies. Last but not least, macrophage-membrane-coated carriers have been investigated with encouraging results.

## 1. Introduction

Atherosclerosis is a complex and multifactorial disease that affects the arterial system, leading to narrowing and obstruction of blood vessels. It is a leading cause of mortality and morbidity worldwide, accounting for the majority of cases of coronary artery disease (CAD). The pathophysiology of atherosclerosis involves multiple mechanisms, including inflammation, lipid deposition, endothelial dysfunction, and smooth muscle cell proliferation [1]. Inflammatory processes play a central role in the initiation, progression, and complications of atherosclerosis [2]. Inflammatory cytokines, such as interleukin-1β (IL-1β), tumor necrosis factor-α (TNF-α), and C-reactive protein (CRP), promote endothelial dysfunction, leukocyte recruitment, and smooth muscle cell migration, proliferation, and apoptosis [2]. The role of inflammation in atherosclerosis and CAD has been supported by numerous epidemiological, genetic, and experimental studies, highlighting the potential of anti-inflammatory therapies for the prevention and treatment of atherosclerotic cardiovascular disease. Inflammatory cells, including monocytes/macrophages, T lymphocytes, and mast cells, infiltrate the arterial wall and release proinflammatory mediators that contribute to plaque rupture and thrombosis.

Macrophages’ role in inflammation and numerous diseases has been extensively investigated. Nevertheless, due to their flexibility and several tissue-specific roles, the full range of their functions has not been explored. Macrophages are detected in practically all tissues and are responsible for maintaining tissue homeostasis, with roles in the phagocytosis of cellular debris and pathogens, tissue repair, and immune response initiation. In the field of atherosclerosis, macrophages are implicated in plaque formation via infiltration to the subendothelial layer in the setting of endothelial dysfunction. Subsequently, the uptake of low-density lipoprotein (LDL) complexes leads to foam cell formation. Apart from plaque formation, macrophages are important contributors to plaque progression via secretion of proinflammatory cytokines and chemokines, as well as extracellular matrix remodeling, which, together with foam cell death and necrosis, leads to fibrous cap thinning and plaque destabilization. Therefore, in this review article, we discuss the importance of macrophages in the initiation and progression of atherosclerosis, while also elaborating on potential approaches to the management of atherosclerosis that involve, either directly or indirectly, targeting macrophages.

## 2. Macrophage Biology and Classification

Macrophages are crucial components of the immune system with diverse functions in both innate and adaptive immunity. Macrophages are present in various tissues throughout the body and act as sentinel cells, surveying their microenvironment for the presence of pathogens and cellular debris. Upon encountering foreign particles, macrophages employ phagocytosis, a process by which they engulf and eliminate pathogens or cellular debris. Additionally, macrophages serve as antigen-presenting cells, playing a pivotal role in initiating adaptive immune responses by presenting antigens to T cells. Furthermore, macrophages secrete a range of cytokines and chemokines that modulate inflammation and recruit other immune cells to the site of infection or injury. The plasticity of macrophages allows them to adopt distinct phenotypes based on environmental cues. Classically activated macrophages M1 exhibit proinflammatory functions, producing cytokines such as TNF-α and IL-1β to combat pathogens. Conversely, alternatively activated macrophages M2 display anti-inflammatory properties and contribute to tissue repair and remodeling. The balance between M1 and M2 macrophages is crucial for maintaining immune homeostasis and effective host defense.

With recent advances in molecular identification techniques, it is now known that there are two main macrophage subtypes: tissue-resident macrophages (TRMs), which are produced early in fetal development, and monocyte-derived macrophages (MDMs), which are the result of monocyte differentiation in the circulation. TRMs and MDMs both have phagocytic and immune response propagation functions, while also facilitating repair and homeostasis. On the contrary, TRMs have the ability to self-renew and take on specific tasks and features depending on the type of tissue in which they reside.

Based on their activation state, macrophages can be broadly classified into two main subtypes: classically activated M1 macrophages and alternatively activated M2 macrophages [3]. M1 macrophages are classically activated by proinflammatory cytokines such as interferon-gamma (IFN-γ) and lipopolysaccharides (LPSs). They produce high levels of proinflammatory cytokines such as IL-1β, interleukin-6 (IL-6), and TNF-α. M1 macrophages also express high levels of inducible nitric oxide synthase (iNOS), which produces high levels of nitric oxide (NO) that contribute to oxidative stress and tissue damage [4]. M1 macrophages are typically located in the shoulder region of the atherosclerotic plaque, where they are exposed to proinflammatory stimuli and contribute to plaque destabilization by promoting matrix degradation and necrotic core formation [5]. M2 macrophages, on the other hand, are alternatively activated by anti-inflammatory cytokines such as interleukin-4 (IL-4) and interleukin-13 (IL-13) [6]. They produce high levels of anti-inflammatory cytokines such as interleukin-10 (IL-10) and transforming growth factor-beta (TGF-β). M2 macrophages also express scavenger receptors such as CD163 and CD206, which are involved in the phagocytosis of apoptotic cells and debris. M2 macrophages are typically located in the core region of the atherosclerotic plaque, where they contribute to tissue repair and resolution of inflammation by promoting fibrosis and angiogenesis. In addition to these two main subtypes, there are also intermediate macrophage phenotypes that can exhibit mixed M1/M2 activation states depending on the local microenvironment. For example, macrophages in the fibrous cap region of the plaque can express both proinflammatory and anti-inflammatory cytokines, suggesting a mixed M1/M2 phenotype. Similarly, macrophages in the adventitia surrounding the plaque can exhibit a reparative phenotype characterized by the expression of markers such as CD206 and TGF-β. Recent studies have identified several new subtypes of macrophages that can be present in atherosclerotic plaques, including Mox, M(Hb), Mhem, M4, and HAMac macrophages (Table 1). These subtypes are characterized by unique gene expression profiles, functional properties, and associations with specific stages of plaque development and progression.

## 3. Atherosclerosis Pathophysiology and the Role of Macrophages

Endothelial dysfunction, abnormal lipid metabolism, and inflammation are the primary causes of atherosclerotic plaque development. There is increased oxidative stress in the presence of genetic vulnerability, cardiovascular risk factors (hypertension, diabetes mellitus, smoking, inflammation), or low shear stress, which promotes endothelial dysfunction, the first stage in atherogenesis [7]. The vascular endothelium is an organ that lines blood vessels and is essential for the formation and progression of atherosclerosis. Endothelial cells are responsible—in a healthy condition—for controlling key activities, such as vascular tone, thrombosis and fibrinolysis, vascular inflammation, and remodeling [8]. NO, which has vasorelaxant, anti-thrombotic, anti-proliferative, and anti-inflammatory characteristics, is the primary regulator of endothelial function. As endothelial cells are stimulated, the phenotypic shifts toward an atheroprone state, with increased vasoconstriction, thrombosis, leukocyte mobilization and migration, and vascular smooth muscle cell (VSMC) proliferation. This is related to decreased NO bioavailability as a result of increased degradation and decreased synthesis. The critical process contributing to impaired NO bioavailability and reactive oxygen species (ROS) production is the uncoupling of endothelial NO synthase (eNOS) as a result of asymmetric dimethylarginine upregulation (endogenous eNOS inhibitor), oxidation of tetrahydrobiopterin (BH4), or failure of the BH4 salvage pathway. Additionally, we should note that the release of inflammatory cytokines by the TRMs, such as TNF-α and IL-1β, promotes the expression of endothelium-derived adhesion molecules and the subsequent entrance of macrophages to the subendothelial layer [9].

Increased permeability promotes the penetration of apolipoprotein-B-containing lipoproteins up to roughly 70 nm in diameter, notably LDL, in the artery wall in the presence of a defective endothelium layer [10]. LDL is a heterogeneous molecule involved in the transfer of insoluble cholesterol. There are four types of LDL, with small-dense LDL (sdLDL) being the most atherogenic. LDL particle inflow is no longer seen as a passive process. LDL is then kept in the vessel wall as a result of the interaction of apoB100’s positively charged arginine and lysine with the negatively charged sulfate and carboxylic acid of the artery wall proteoglycans. TRMs manage lipids in the extracellular space through intracellular metabolic processes (such as autophagy), storage (via lipid droplet production), or export (via free cholesterol transfer to available HDL particles) under homeostatic settings. Yet, there is a lack of cholesterol control as atherosclerosis progresses. LDL can then be modified in a variety of ways, including oxidation, electronegativity, desyalation, glycation, and self-association [11]. Oxidized LDL (oxLDL) molecules link to the lectin-like oxLDL receptor-1 (LOX-1) on the surface of VSMCs and macrophages, causing foam cells to develop [12]. Moreover, LDL particles can form complexes with the artery wall’s proteoglycans and glycosaminoglycans, which can be taken up by macrophages.

Opposing actions are performed by HDL, which is responsible for cholesterol efflux. However, when HDL is dysfunctional or found in low concentrations, or when autophagy is impaired, endoplasmic reticulum stress prevails [13,14]. This leads to foam cell apoptosis or necroptosis. The protective mechanisms include efferocytosis by other macrophages, which is, however, dampened in advanced atherosclerosis. As a result, excessive necroptosis through the receptor-interacting serine/threonine-protein kinase 1 (RIPK1), RIPK3, and mixed-lineage kinase domain-like pseudokinase (MLKL) cascade provokes the enhanced release of proinflammatory molecules, which represent a signal for further mobilization and migration of MDMs [15,16]. The presence of CD47, along with the downregulated expression of MerTK, is an additional contributor to deficient efferocytosis [17]. Necroptosis is not the only form of cell death noted in atherosclerotic plaques, with pyroptosis (inflammatory cell death due to caspase-1) and ferroptosis (inflammatory cell death due to iron-dependent lipid peroxidation) being equally important [18,19]. Overall, the combination of excessive cell death with the impaired ability to clear cellular debris leads to the formation of a lipid-rich necrotic core, which is often considered a hallmark of plaque instability.

The alterations in extracellular matrix (ECM) components are another mediating factor in the progression of atherosclerosis. The ECM of atherosclerotic plaques clearly contributes to the pathogenesis of atherosclerosis through dynamic changes in the balance of its components [20]. The ECM can impact a variety of activities, including cell migration, progenitor cell self-renewal and differentiation, tissue development and morphogenesis, and fibrosis. In reality, elastin is the primary ECM ingredient in healthy arteries, transitioning to proteoglycans in early atherosclerosis lesions and finally collagen in late atherosclerotic lesions. ECM components are largely secreted by regulated VSMCs in the subendothelial region, whereas matrix metalloproteinases (MMPs), which break down the ECM and other proteins, are primarily produced by macrophages. The ECM/MMP ratio is carefully controlled and responsive to inflammatory stimuli such as TGF-β, boosting collagen and elastin formation. Tissue inhibitors of metalloproteinases (TIMPs) are also released as endogenous inhibitors by those same cells, allowing for rapid modulation of MMP-mediated ECM remodeling [21]. TGF-β is generated by endothelial cells or other cells and stimulates collagen synthesis, but proinflammatory TNF-α and IFN-γ released by inflammatory cells inhibit collagen synthesis [21]. It thus becomes clear that the interplay of macrophages with VSMCs is crucial in ECM remodeling.

The interaction of macrophages with other cell types in the atherosclerotic plaque should not go unnoticed. Since macrophages are antigen-presenting cells, they display oxLDL as a foreign substance on their major histocompatibility complex to activate T lymphocytes, further promoting B-cell activation [22]. M1 macrophages and Th1 cells contribute to atherosclerosis by secreting proinflammatory cytokines and chemokines, whereas M2 macrophages, Tregs, and B-1 cells control inflammation, reduce plaque size, and enhance plaque stability. While macrophage recruitment and interactions with other cells in the artery wall directly induce atherosclerosis, they are also required for plaque stability and retreat. The process of atherosclerosis regression involves newly recruited monocytes from the circulation in response to CCR2, since regression does not occur without these macrophages [23]. MDMs that are unable to become inflammatory macrophages, on the other hand, can be driven toward an anti-inflammatory phenotype to promote atherosclerosis regression [24]. The principal method by which existing atherosclerotic lesions begin to shrink and resolve, both clinically and experimentally, is by reducing circulating plasma lipids. In animal models, this is frequently followed by an increase in cholesterol efflux from foam cells via ATP-binding cassette transporter (ABCA)1 to apoA1/HDL via the reverse cholesterol transport route [25]. When cholesterol efflux is induced in high HDL environments, plaque macrophages adopt a pro-resolving M2-like phenotype, producing anti-inflammatory cytokines such as IL-10 and TGF-β, supporting tissue repair and angiogenesis. The pro-resolving phenotype also enhances debris phagocytosis and apoptotic cell efferocytosis, which aid in the reduction in the necrotic core. Indeed, efferocytosis and apoptotic cell destruction enhance macrophage proliferation, increasing the number of macrophages accessible for efferocytosis and thereby boosting the regression process.

## 4. The Effect of Anti-Atherosclerotic Pharmacotherapy on Macrophages

The impact of current anti-atherosclerotic medication on macrophage polarization and function has been examined in preclinical studies. Among contemporary therapies, statins have been established as a core treatment approach due to their lipid-lowering effect, as well as their pleiotropic actions. Zhang et al. examined the effect of pravastatin on ApoE^−/−^ mice and found a reduction in the ratio of M1/M2 macrophages in atherosclerotic plaques, despite an increase in aortic plaque size [26]. This may signify an underlying mechanism of statin-induced plaque stabilization, since M2 macrophages are the predominant subtype at the phase of plaque regression [27]. Moving to Proprotein convertase subtilisin/kexin type 9 (PCSK9) inhibitors, alirocumab improved plaque stability by decreasing the plaque macrophage content, further enhancing the effect of atorvastatin in APOE*3Leiden [28]. In humans with an acute coronary syndrome, the addition of evolocumab on top of rosuvastatin decreased the macrophage grade and increased fibrous cap thickness compared to statin treatment alone [29]. Similarly, in patients with non-ST-segment elevation myocardial infarction, the macrophage index—which is defined as the product of the average macrophage arc and length of the lesion with macrophage infiltration—was significantly lower in patients treated with evolocumab compared to the placebo [30]. Novel antidiabetic agents, namely Glucagon-like peptide-1 agonists that have proven cardiovascular benefits, may also promote macrophage polarization towards an M2 phenotype, as shown in studies of exenatide and lixisenatide [31,32]. Finally, sodium-glucose cotransporter-2 inhibitors may affect macrophage polarization, as shown in recent mechanistic studies. Koyani et al. demonstrated the M2 polarization of lipopolysaccharide-treated macrophages through the expression of relevant markers (reduced CD40 and increased CD206 staining) [33]. Empagliflozin reduced M1-polarized macrophage accumulation while inducing the anti-inflammatory M2 phenotype of macrophages in a study by Xu et al. [34]. Moreover, dapagliflozin also attenuated the percentage of M2 macrophage infiltration [35].

## 5. Targeting Macrophages for Atherosclerosis Treatment

Based on the evidence provided above, it becomes clear that macrophages could represent an appealing therapeutic target in the setting of atherosclerotic diseases through several mechanisms (Table 2). To begin with, since M1 and M2 macrophages have opposing actions with regard to inflammation, the alteration in macrophage polarization from an M1 to an M2 phenotype could positively influence atherosclerotic plaque progression. Although the available data are scarce, a few studies have detected such effects. Li et al. found that crocin, an active ingredient of Crocus sativus L, induced a macrophage phenotypic switch towards an M2 phenotype in a rat model of coronary atherosclerosis by targeting the 5′ AMP-activated protein kinase (AMPK) pathway [36]. Ginsenoside Rb1, a core compound of Panax ginseng, led to augmented expression of M2 macrophage markers and downregulated expression of M1 macrophage markers, which were also accompanied by atherosclerotic plaque stabilization in ApoE^−/−^ mice [37]. Similar findings were reported in diabetic atherosclerotic mouse models [38]. Moving to geniposide, its administration in New Zealand rabbits on a high-fat diet led to the polarization of intraplaque macrophages toward an M2 phenotype, with subsequent attenuation of atherosclerotic lesion burden in aortic tissue [39]. Moreover, the flavonol protocatechuic acid promoted the M2 macrophage phenotype in ApoE^−/−^ mice through the phosphoinositide 3-kinase (PI3K)-Akt-mediated nuclear-factor-κB pathway inhibition, signal transducer and activator of transcription (STAT)6 phosphorylation, and peroxisome Proliferator-Activated Receptor (PPAR)γ activation [40].

Non-lethal sonodynamic therapy has also proved efficacious in promoting a beneficial macrophage phenotypic switch of bone marrow and TRMs, as shown in the experimental study of Yang et al. in atherosclerotic models [41]. The AMPK-mammalian target of the rapamycin complex 1 (mTORC1) pathway was found to be responsible for these alterations, signifying the importance of macrophage autophagy [41]. Autophagy is the process by which cells digest themselves in order to preserve cell homeostasis and destroy mistranslated proteins and organelles. Autophagy is important in the early stages of atherosclerosis in removing cholesterol deposits in blood vessels [51]. The deletion of key autophagy-related genes (Atg5, Atg7, Atg14) in macrophages speeds up the development of atherosclerotic plaques in mice, and macrophage-specific Atg5 or Atg14 knockout animals had greater plaque apoptosis and bigger necrotic cores [51,52]. Macrophage autophagy can be employed as a possible treatment option for atherosclerosis since it plays an important protective function in the course of atherosclerosis. Beginning with trehalose, it was able to induce autophagy and lysosomal biogenesis in ApoE^−/−^ mice [42]. Hypericin and cordycepin may also affect key autophagy pathways, namely AMPK-mTOR, as seen in experimental studies [43,44]. The promotion of autophagy through sirtuin-1 via the administration of resveratrol and berberine was successful, according to other reports [45,46]. Arsenic trioxide acted through the PI3K/Akt/mTOR pathway to upregulate the process of autophagy in ApoE^−/−^ mice [47]. Another approach focuses on nanoparticles to promote autophagy. Most nanoparticles are taken up non-selectively by the mononuclear monocytes/macrophages, resulting in undesired destruction in lysosomes. A recent study tried to take advantage of this hypothesis in order to target macrophage lysosomes. The administration of two commonly used acidic nanoparticles, poly-lactide-co-glycolic acid and polylactic acid, enhanced lysosomal degradation, promoted macroautophagy/autophagy and protein aggregate removal, and reduced apoptosis and inflammasome activation [48]. Ultimately, the administration of these acidic nanoparticles had significant anti-atherosclerotic effects, including attenuated necrotic core formation and fibrous cap thinning. Finally, the modulation of microRNAs could be crucial in the regulation of macrophage autophagy. Wang et al., in a study on ox-LDL-induced THP-1 macrophages, found that miR-99a-5p overexpression inhibited NACHT, LRR, and PYD domains-containing protein 3 (NLRP3) inflammasome activation and stimulated macrophage autophagy through mTOR, producing a lower atherosclerotic lesion burden [49]. Shao et al. treated apoE^−/−^ mice and RAW264.7 cells with miR-29a mimic, and found polarization of macrophages toward an M2 phenotype, along with overexpression of autophagy-related proteins [50]. Additionally, inhibition of miR-33 upregulated macrophage autophagy in the study of Ouimet et al. [53].

Other than a direct effect on macrophage phenotype and autophagy, macrophages could act as targets for drug delivery in the setting of atherosclerosis, indirectly affecting its progression. Macrophages express a variety of protein receptors, including the mannose receptor, macrophage scavenger receptor (SR), CD44 receptor, and folate receptor, which can be employed as possible therapeutic targets [54]. Mannose receptor is a C-type lectin found on macrophage surfaces. Mannose-functionalized nanoparticles are preferentially uptaken by macrophages in atherosclerotic plaques [55]. The targeted administration of the LXR agonist T0901317 dramatically lowers the plaque area and MMP-9 expression in the animal aortic arch [55]. T0901317, an LXR agonist, reduces atherosclerosis, although side effects include hepatic steatosis and hypertriglyceridemia [56]. As a result, a new platform known as D-Nap-GFFY encapsulating T0901317 nanofiber hydrogel is being developed to target macrophages at atherosclerosis sites and minimize lesions while having no effect on hepatic lipogenesis [57]. Recently, Song et al. developed a macrophage-mannose-receptor-targeted nanodrug (mannose-polyethylene glycol-glycol chitosan-deoxycholic acid-cyanine 7-lobeglitazone; MMR-Lobe-Cy) that can detect plaque inflammation that is accompanied by high concentrations of macrophages and consequently mannose receptors [58]. This nanodrug delivered lobeglitazone at those vulnerable sites, leading to attenuated inflammation and promotion of plaque stabilization due to decreased macrophage content and increased collagen deposition.

CD44 is a cell surface receptor that is overexpressed on cells in atherosclerotic plaques and is required for plaque development. The CD44 protein is abundantly expressed in atherosclerosis macrophages [59], demonstrating that CD44 can be exploited as a target of atherosclerosis macrophages. The CD44 receptor’s natural ligand is hyaluronic acid (HA). Magnetic nanoparticles made of iron oxide and coated with HA-target-activated macrophages for macrophage imaging. LOX-1-specific siRNA nanocomposites coated with HA overcome siRNA’s intrinsic constraints, such as its negative charge, high molecular weight, instability, and target siRNA delivery to macrophages. It was proven that LOX-1 siRNA could lower LDL absorption by macrophages, slowing the course of atherosclerosis [60]. The combination of conventional atherosclerosis treatments with HA could thus represent an appealing approach. Specifically, atorvastatin is an important lipid-lowering medication, although its use is restricted due to its hydrophobicity. A novel HA–atorvastatin (ATV) combination can protect the drug’s hydrophobicity while also targeting atherosclerotic macrophages [61]. In vitro investigations have revealed that HA-ATV conjugates have a high affinity for macrophages [61]. HA-ATV nanoparticles effectively decreased inflammation in advanced atherosclerotic plaques after one week of dosing [61]. A mechanically sensitive macrophage-targeted HA hydrogel carrier has recently been developed to deliver simvastatin to treat atherosclerosis [62]. The scavenger receptor Stabilin-2, the main clearance receptor for HA, was the target of DSPE-PEG liposomes loaded with CaMKIIγ siRNA and modified with P2P polypeptide [63]. This molecule could inhibit macrophage CaMKIIγ, resulting in endocytosis, increased fibrous cap thickness, and attenuated necrotic core formation [64].

As far as macrophage folate receptors are concerned, they have been utilized in atherosclerosis imaging in the past as a marker of macrophage activation [65]. By exploiting the abundance of folate receptors, Fang et al. administered folate-modified liposomes loaded with telmisartan in an attempt to specifically deliver this drug in atherosclerotic plaques of ApoE^−/−^ mice. Under this treatment, a regression of atherosclerotic plaques was noted, possibly due to the ameliorated cholesterol efflux, fibrous cap thickening, and attenuated macrophage invasion [66]. Furusho et al. had previously shown that a recombinant immunotoxin consisting of the truncated Pseudomonas exotoxin A conjugated to an anti-folate receptor β antibody could reduce the number of macrophages in ApoE^−/−^ mice, thus halting atherosclerosis progression [67]. Additionally, folate-targeted liposomes carrying betamethasone reduced the number of folate-receptor-positive cells in an atherosclerotic mouse model [68].

Finally, in atherosclerosis, the class B scavenger receptors CD36 and A (SR-A) are primarily responsible for the absorption of modified LDL by macrophages. Since phosphatidylserine can selectively target macrophage CD36 receptors, a dual-function nano-platform employing phosphatidylserine to modify recombinant high-density lipoproteins was loaded with pitavastatin peroxidase and SR-A siRNA. SR-A siRNA silenced the expression of the SR-A receptor in macrophages, increasing the expression of the CD36 receptor through positive feedback regulation and increasing macrophage uptake of nanoparticles [69]. Long-term administration of this compound decreased the atherosclerotic plaque area and macrophage accumulation [69]. ATV-loaded phosphatidylserine liposomes also enhanced cholesterol efflux and impaired adhesion molecule expression in New Zealand rabbits fed a high-fat diet [70]. In a study by Wu et al., an apoptotic body biomimic liposome, constructed by integrating phosphatidylserine and DSPE-PEG2000-cRGDfK onto the surface of the liposome, was used to selectively deliver pioglitazone to macrophages in vitro [71]. This resulted in greater endothelial invasion and attenuated plaque progression by upregulation of anti-inflammatory macrophages and cytokines [71]. Curcumin-loaded phosphatidylserine-containing nanostructured lipid carriers were effective in preventing lipid accumulation and inflammatory activation of cultured macrophages [72]. The effect was greater when compared to curcumin or phosphatidylserine alone, further highlighting the potential of selective drug delivery to macrophages in the management of atherosclerosis.

Perhaps an even more appealing approach is the biomimetic camouflaging of macrophage-membrane-coated carriers, which can protect the drug carrier from clearance by immune cells while also partially retaining the ability to invade atherosclerotic lesions. Such macrophage-membrane-coated carriers could be superior to the nanoparticles mentioned above due to their longer retention time and lower immunogenicity. Leukosomes consist of leukocyte plasmalemma proteins in a synthetic phospholipid bilayer and contain important surface proteins that are necessary for vascular invasion [73]. Importantly, when loaded with anti-atherosclerotic molecules, such as rapamycin, a reduction in macrophage proliferation can lead to plaque stabilization [73]. Nanoparticles coated with macrophage cell membrane constituents can also bind cytokines and inhibit the inflammatory cascade, as they contain cytokine receptors (CD126, CD130, CD120, CD119) [74]. Last but not least, molecularly engineered M2 macrophage exosomes, electroporated with hexyl 5-aminolevulinate, produced antioxidant and anti-inflammatory effects and induced an M1 macrophage phenotypic switch [75].

## 6. Conclusions

Macrophages represent key orchestrators of the atherosclerotic process by regulating cholesterol uptake, foam cell formation, and propagation of inflammation, among other functions. Macrophages in atherosclerotic plaques can be classified into different subtypes based on their activation state and location within the plaque. Understanding these different subtypes and their roles in plaque progression and regression can provide insights into the underlying mechanisms of atherosclerosis and may lead to the development of new therapeutic strategies for this disease. Existing pharmacological options in atherosclerotic diseases have also proven their pleiotropic effects in this field due to their efficacy in attenuating macrophage infiltration and promoting macrophage polarization towards an M2 phenotype. Specific macrophage manipulation, either via polarization toward an M2 phenotype or induction of macrophage autophagy, has shown promise in preclinical studies and warrants further investigation. Indirectly manipulating macrophage receptors for drug targeting and the use of macrophage-membrane-coated carriers could emerge as novel appealing approaches to the management of atherosclerotic diseases. Last but not least, the identification of new subtypes of macrophages in atherosclerotic plaques highlights the complexity of macrophage biology in this disease and may provide new targets for therapeutic intervention. Further research is needed to fully understand the roles of these subtypes in plaque development and to determine their potential as biomarkers or therapeutic targets in atherosclerosis.

## Figures and Tables

**Table 1:** Novel macrophage phenotypes and their role in atherosclerosis.

Macrophage Phenotype	Characteristics	Effect in Atherosclerosis	Implications
Mox macrophages	Alternatively activated M2 macrophages High expression of heme oxygenase-1 and oxidative stress response genes	Promote heme detoxification Reduce oxidative stress Inhibit foam cell formation	Enriched in stable plaques Associated with lower risk of adverse cardiovascular events
M(Hb) macrophages	Alternatively activated M2 macrophages High expression of haptoglobin and hemoglobin scavenging receptors (CD163, CD163L1)	Scavenge free hemoglobin and prevent its pro-oxidative effects	Enriched in advanced plaques Associated with plaque vulnerability
Mhem macrophages	Alternatively activated M2 macrophages High expression of hemoglobin alpha and beta chains and heme transporters such as CD163 and heme carrier protein 1	Promote erythrocyte turnover by phagocytosing senescent and damaged erythrocytes, and recycle their iron and heme	Enriched in early plaques Associated with a reduced risk of cardiovascular events
M4 macrophages	Subtype of classically activated M1 macrophages High expression of chemokines such as CCL2 and CXCL4 and proteases such as MMP-12	Recruit monocytes and neutrophils Degrade extracellular matrix proteins	Enriched in unstable plaques Associated with a higher risk of cardiovascular events
HAMac macrophages	Subtype of classically activated M1 macrophages High expression of proinflammatory cytokines such as IL-1β, IL-6, and TNF-α, and low expression of markers of alternative activation such as CD206 and TGF-β	Hemophagocytosis Produce high levels of proinflammatory cytokines and induce apoptosis of smooth muscle cells	Enriched in unstable plaques Associated with a higher risk of cardiovascular events

CCL2: C-C Motif Chemokine Ligand 2, CXCL4: C-X-C motif ligand 4, MMP: matrix metalloproteinase, IL: interleukin, TNF: tumor necrosis factor, TGF: transforming growth factor.

**Table 2:** Experimental studies assessing therapeutic approaches directly targeting macrophages.

Study	Agent	Experimental Model	Findings
Li et al. [36]	Crocin	Rats with coronary atherosclerosis	Phenotypic switching (M1 to M2 macrophages)
Zhang et al. [37]	Ginsenoside Rb1	ApoE^−/−^ mice	Increased expression of M2 macrophage markers Reduced expression of M1 macrophage markers Atherosclerotic plaque stabilization
Jin et al. [39]	Geniposide	New Zealand rabbits on a high-fat diet	Intraplaque macrophage polarization toward an M2 phenotype Attenuation of aortic atherosclerosis
Liu et al. [40]	Protocatechuic acid	ApoE^−/−^ mice	Promotion of an M2 macrophage phenotype
Yang et al. [41]	Non-lethal sonodynamic therapy	Atherosclerosis	Phenotypic switching of bone-marrow- and tissue-resident macrophages Macrophage autophagy
Evans et al. [42]	Trehalose	ApoE^−/−^ mice	Induction of macrophage autophagy and lysosomal biogenesis
Li et al. [43]	Hypericin	ApoE^−/−^ mice	Modulation of the AMPK-mTOR autophagy pathways
Li et al. [44]	Cordycepin	THP-1 macrophage	Modulation of the AMPK-mTOR autophagy pathways
Liu et al. [45]	Resveratrol	ox-LDL-induced apoptotic RAW264.7 cells	Promotion of autophagy through sirtuin-1
Zheng et al. [46]	Berberin	Peritoneal macrophages	Promotion of autophagy through sirtuin-1
Fang et al. [47]	Arsenic trioxide	ApoE^−/−^ mice	Upregulation of autophagy through the PI3K/Akt/mTOR pathway
Zhang et al. [48]	Poly-lactide-co-glycolic acid Polylactic acid	Macrophages	Increased lysosomal degradation Enhanced autophagy Reduced apoptosis and inflammasome activation Attenuated necrotic core formation and fibrous cap thinning
Wang et al. [49]	MiR-99a-5p	ox-LDL-induced THP-1 macrophages	Inhibition of NLRP3 inflammasome activation Macrophage autophagy Diminished atherosclerotic lesion burden
Shao et al. [50]	MiR-29a mimic	ApoE^−/−^ mice RAW264.7 cells	Macrophage polarization toward an M2 phenotype Overexpression of autophagy-related proteins

AMPK: 5′ AMP-activated protein kinase, mTOR: mammalian target of rapamycin, ox-LDL: oxidized low-density lipoprotein, PI3K: phosphoinositide 3-kinase, NLRP3: NACHT, LRR, and PYD domains-containing protein 3.

## Data Availability

Not applicable.
